# Supplementary material for: Prechtl’s method to assess general movements: Inter-rater reliability during the preterm period
Source: PLoS One. 2024 Apr 18;19(4):e0301934. doi: 10.1371/journal.pone.0301934 (PMC11025967; doi:10.1371/journal.pone.0301934)
Supplement: Attachment — Submitted filename: Response to Reviewers.docx [file pone.0301934.s002.docx]

Angélica VALENCIA

Faculty of Psychology

University Cooperativa of Colombia

December 01, 2023

Claudia BROGNA
Academic Editor
PLOS ONE

Dear Dr. BROGNA,

We appreciate the Academic Editor and Reviewers' feedback on our manuscript and respond to each point raised:

- **Academic Editor: "Please ensure that your manuscript meets PLOS ONE's style requirements, including those for file naming."**

We have revised our manuscript to ensure full compliance with PLOS ONE's style for authors' affiliations, headings, tables, and reference citations. Each file is now named according to the requirements, which includes 'Response to Reviewers', 'Revised Manuscript with Track Changes', 'Manuscript', and 'Supporting Information'.

- **Academic Editor: "In your Data Availability statement, you have not specified where the minimal data set underlying the results described in your manuscript can be found. PLOS defines a study's minimal data set as the underlying data used to reach the conclusions drawn in the manuscript and any additional data required to replicate the reported study findings in their entirety. All PLOS journals require that the minimal data set be made fully available."**

To address this request, we have uploaded our study’s minimal underlying data set as 'Supporting Information' as an Excel file upon submitting our revised manuscript.

- **Reviewer #1: " The discussion is not clearly organized. It is difficult to understand the main points the authors are trying to make."**

In response, we have restructured the discussion (page 11, line 262). The discussion now addresses three main results: inter-rater reliability for the global and detailed assessments of general movements (GMs) using the Prechtl' method (GMA) during the preterm period, as well as the precision of these estimates.

Firstly, we addressed the differences in inter-rater reliability for the global assessment (page 12, line 274). Since the GMs classification with abnormal subcategories obtained lower inter-rater reliability compared to the binary GMs classification, we discussed the factors that might have influenced this difference.

Secondly, we discussed the factors that might have influenced the inter-rater reliability of the detailed assessment because the observed inter-rater reliability for the GMOS (GMs optimality score) contradicted our hypothesis and previous research (page 13, line 300).

Thirdly, we discussed the potential impact of small sample size and variability on the precision of our estimates (page 14, line 326). This was essential because we observed wide 95% confidence intervals associated with our inter-rater reliability index for both the global and detailed assessments.

- **Reviewer #1: "The authors identified 95% confidence intervals that included much lower values for reliability. The authors indicated this may affect the generalization of the results. However, the confidence interval would be likely to narrow with larger sample sizes and given their small number of participants, it is not clear if they have proven their conclusion". "The conclusions are somewhat vague. It is difficult to understand the main points the authors are trying to make".**

We agree with Reviewer #1 and have updated the conclusion (page 15, line 367). The revised conclusion no longer focuses on problems related to the generalization of results. Instead, it highlights concerns about the precision of estimates due to the wide confidence intervals observed (Gardner and Altman, 1986). The conclusion is now more precise, addressing the potential impact of our small sample size on the width of the confidence intervals, which, in turn, can affect the precision of our inter-rater reliability estimates. Additionally, the conclusion discusses how challenging items and differences among raters might influence inter-rater reliability for the preterm GMA.

- **Reviewer #2: "Introduction Pag 9 line 57: I’m not sure that the GMs should be considered a “neuromotor test”. It measures the quality of movement detecting disorders of movement (functional limitation), as better explained few lines after."**

We agree with Reviewer #2 and have used the accurate terminology. The revised introduction (page 3, line 64) defines the GMA as a test for the functional assessment of the young nervous system (Einspieler and Prechtl, 2005).

- **Reviewer #2: " It could be of interest to know more information about the clinical characteristics in term of risk of neurological impairment; for example, the US scan data or MRI, seizures, incidence of SGA or other potential risk factors"**

In response, we have included an additional table (Table 2 in page 9, line 225) in the results section, which provides information on participants' clinical characteristics in term of risk of neurological impairment. It includes the US scan, incidence of SGA, APGAR at 5 min, extremely low birth weight, and neonatal clinical complications.

- **Reviewer #2: "please revise all the manuscript for English"**

In response, we have requested professional language editing services to enhance the quality of English in our manuscript. We uploaded the certification of proofreader.

Once again, we thank your invaluable comments, which have undoubtedly improved the quality of our manuscript. We look forward to your decision regarding the revised submission.

Sincerely,

Angélica VALENCIA
