# Supplementary material for: Prechtl’s method to assess general movements: Inter-rater reliability during the preterm period
Source: PLoS One. 2024 Apr 18;19(4):e0301934. doi: 10.1371/journal.pone.0301934 (PMC11025967; doi:10.1371/journal.pone.0301934)
Supplement: Attachment — Submitted filename: Response to Reviewers.docx [file pone.0301934.s003.docx]

Angélica VALENCIA

Faculty of Psychology

University Cooperativa of Colombia

March 11, 2024

Claudia BROGNA
Academic Editor
PLOS ONE

Dear Dr. BROGNA,

We appreciate the Academic Editor and Reviewers' feedback on our manuscript and respond to each point raised:

**Academic Editor: "** **Please review your reference list to ensure that it is complete and correct. If you have cited papers that have been retracted, please include the rationale for doing so in the manuscript text, or remove these references and replace them with relevant current references. Any changes to the reference list should be mentioned in the rebuttal letter that accompanies your revised manuscript. If you need to cite a retracted article, indicate the article’s retracted status in the References list and also include a citation and full reference for the retraction."**

We have careful reviewed our reference list. It is complete, accurate, and does not include any retracted papers. We have added four references.

- Reference 26 (Crowle C, et al., 2017) on page 8, line 201 provides further justification for using the AC1 reliability coefficient, as recommended by Reviewer 3.
- References 32 (Ment LR, et al., 2012), 33 (de Vries L, Eken P, and Dubowitz L, 1992), and 34 (Hadchouel A, and Delacourt C, 2013) on page 9, line 228, define the participants morbidities presented in Table 2, as recommended by Reviewer 1.

**Reviewer #1: "A table of neonatal morbidities has been added but these should be defined in the text."**

In response, we have presented the criteria for defining the following morbidities: small for gestational age, brain ultrasound abnormalities, intraventricular hemorrhage (IVH), periventricular leukomalacia (PVL), and bronchopulmonary dysplasia (Note in Table 2, page 9, line 228).

We have removed the prevalence of respiratory distress and infection (Table 2, page 9, line 228) due to missing data regarding the definition of these morbidities.

**Reviewer #1: "The rate of PVL seems unusually high and this may reflect the definition of PVL or selection of patients for the study."**

We agree with Reviewer #1 and have added (page 15, line 365) that the high rate of PVL in our sample may be explained by the convenience recruitment of high-risk infants admitted to the NICU, as stated in participants section (page 5, line 141).

**Reviewer #3: " Would be good to use the term “preterm writhing GMA” to add clarity about which period of GMs the study focuses on. Similarly, the term “preterm writhing GMA” does not appear anywhere in the abstract. This is important to highlight to differentiate from fidgety GMs".**

**"Ideally throughout the manuscript, the distinction between fidgety and writhing should be made to avoid any confusion. E.g. when discussing previous literature about interrater agreement and predictive validity – are the studies referenced in relation to writhing or fidgety movements? Sometimes it is qualified in the manuscript, and sometimes it is not".**

We have implemented the suggestion from Reviewer #3 and updated the abstract (page 2, line 35) accordingly. The abstract now uses the term "preterm writhing GMA". This term has been used throughout the entire text to enhance clarity and precision in terminology throughout the manuscript.

Additionally, the updated introduction (page 4, lines 90-95) now distinguishes between writhing GMA and fidgety GMA while summarizing previous literature on interrater agreement and predictive validity.

**Reviewer #3: Avoid definite statements, e.g. “…has not been reported”; “this is the first study”. There may be conference abstracts or similar that details this. Instead, consider using “little/scarce publish data exists” or “to our knowledge, few studies report…”/”there is a scarcity of data..”**

We have replaced definite statements throughout the entire text, specifically in the abstract (page 2, line 33), introduction (page 4, lines 101 and 106), and discussion (page 11, line 263), using the nuanced statements (e.g., there is a scarcity of data) recommended by Reviewer #3.

**Reviewer #3: The authors do well to describe the gap in knowledge (although some expressions could be less definitive, see above), but justification for the importance of the knowing the accuracy in the preterm writhing period is lacking. i.e. what is the clinical benefit of having this knowledge during the preterm period? This point may be able to be addressed easily by highlighting research about preterm writhing movements, specifically, and their relationship with later development (line 92 – unclear if this research refers to preterm writhing or fidgety GMA, best to check).**

We agree with Reviewer #3 and have updated the justification (page 4, line 113-118) about the clinical importance of understanding the reliability of the preterm writhing GMA. It now includes the following arguments:

- The demonstrated high validity of the preterm writhing GMA in predicting development in preterm infants (Craciunoiu O, and Holsti L. 2016)
- The value of this information to guide clinicians about consistency of the preterm writhing GMA in identifying at-risk babies and opportunities for early interventions.

Additionally, we have clarified that the validation study cited on page 2, line 92 refers to preterm writhing GMA.

**Reviewer #3: "Crying and hiccupping episodes were suppressed with an online editing program” – I’m not sure I understand how this is possible? Which program was used? Perhaps this program needs to be quoted, as you would with statistical analysis software. This sounds like a very complex procedure and could affect how general movements are observed with human Gestalt perception. Do the authors perhaps mean they were simply edited out?"**

Yes, we mean that the videos from the participants were edited. Therefore, the updated version of the method section (page 6, line 157) simply states this.

**Reviewer #3: "The authors may want to refer to the work by Dr Crowle Early Human development, 104, 2017, to further justify the use of Gwets AC1."**

In response, we have included the reference of Crowle C, et al. (2017) in the statistical analysis section (page 8, line 201)

Once again, we thank your invaluable comments, which have undoubtedly improved the quality of our manuscript. We look forward to your decision regarding the revised submission.

Sincerely,

Angélica VALENCIA
